# “It means you are grounded” – caregivers’ perspectives on the rehabilitation of children with neurodisability in Malawi

**DOI:** 10.3109/09638288.2015.1035458

**Published:** 2015-04-16

**Authors:** Amelia Paget, Macpherson Mallewa, Dorothy Chinguo, Chawanangwa Mahebere-Chirambo, Melissa Gladstone

**Affiliations:** ^a^ Centre for Child and Adolescent Health, School of Social and Community Medicine, University of Bristol, Bristol, UK; ^b^ College of Medicine, University of Malawi, Blantyre, Malawi; ^c^ Brain Infections Group, Institute of Infection and Global Health, University of Liverpool, Liverpool, UK; ^d^ Queen Elizabeth Central Hospital, Blantyre, Malawi; ^e^ Malawi Liverpool Wellcome Trust, Blantyre, Malawi; ^f^ Department of Women’s and Children’s Health, Institute of Translational Medicine, University of Liverpool, UK

**Keywords:** Africa, carers, child, developing countries, disability, qualitative, rehabilitation

## Abstract

*Purpose*: Rates of childhood disability are estimated to be high in African settings; however, services to provide information and support are limited. This study aims to explore perspectives and experiences of caregivers of children with disabilities (CWD) from acquired brain injury to inform the development of training packages for health-workers (HW) in hospital settings. *Methods*: The study was conducted in a tertiary hospital using qualitative methods. Fourteen in-depth interviews (IDIs) were conducted with parents/carers (PC), and 10 IDIs and 4 focus-group discussions (FGDs) with HW. Data were audio-recorded, transcribed, translated and analysed using thematic approaches. *Results*: HWs and PCs held varying perspectives on aetiology and prognosis for CWD. HWs raised concerns about impact on families, risks of neglect and abuse. Barriers to care and support included prioritisation of acute illness, lack of HW knowledge and confidence, stigma, poor communication, focus on physical disability, and poor availability of services. Among ideas for improvement, good communication and counselling was seen as a priority but not often achieved. *Conclusion*: A range of family, health service and wider contextual factors affect care for CWD. Training for HW should emphasise disability rights, access to services, a range of disabilities and specific training on counselling.

**Implications for Rehabilitation**
- To create good training programmes for workers who manage children with neurodisability, workers’ views on their training needs, as well parents’ views of what feel they need to know most, must be taken into account.
- The need for training regarding communication skills is a priority for health-workers (HW), who manage children with neurodisability in Malawi and confidence in this area is likely to be vital in providing support for these families.
- Disability rights and inclusion should be imperative in any training programme for managing children with neurodisability in hospital settings.
- Even in low-resource settings such as Malawi, it is vital that the links between hospital management and the limited resources in the community are updated and maintained.

## Background

The State of the World’s Children report provides a shockingly high estimate of 1 in 20 children under the age of 14 years having a disability. Furthermore, 75% of those with a disability are estimated to live in low- and middle-income countries [1]. Neurodisability, an umbrella term for a number of conditions associated with an impairment of the nervous system causing functional limitation [2], is the most common cause of disability in children. With improvements in child survival and with the majority of children with disabilities (CWD) worldwide living in low- and middle-income (LAMI) countries [3], disability in childhood is an emerging global health priority [4]. Research on childhood neurodisability in low- and middle-income countries (LMIC) so far however, has mainly focused on epidemiological studies with limited research on the provision of effective interventions [5,6].

Many African countries, Malawi being one of these, have particularly high rates of childhood neurodisability, with much of this being caused by illnesses such as meningitis, cerebral malaria, and encephalitis, but also by trauma and birth asphyxia [7–10]. Recent key informant studies in Malawi have provided rates of childhood disability of 17/1000 with neurodisability affecting the majority of children (cerebral palsy, cognitive problems, epilepsy, visual and hearing problems) [11]. Malawi ranks within the lowest 20 countries in the human development index, has 50% of the population living below the poverty line of $1 per day and although improving, still has high rates of fertility with over 5 births per woman and high rates of under-five mortality [12].

In settings such as Malawi, there is often a strong focus on the prevention and acute management of childhood illnesses, rather than the consequences. Malawi has one medical school as well as two major paediatric units and a number of smaller general hospitals. Community-based rehabilitation has been present in the country since 1987 but training programmes are limited and under review [13]. Services and support for children are often scarce [14] with only a handful of occupational therapists, speech and language therapists and physiotherapists throughout the country. Many children with neurodisability in such settings face a daily reality of exclusion and no access to rehabilitative health care or support services, and a significant proportion are unable to acquire a formal education [15]. Services that do exist are often largely concentrated in urban centres or provided by non-governmental organisations. With the limited resources available within this setting, concentrating first on the training and support of those within the paediatric units is a crucial step to undertake.

In order to provide appropriate support for children with neurodisability in LAMI settings, effective programmes and training of professionals need to be established and integrated with existing services. Evidence is lacking on how best to design, implement and evaluate such programmes at a local level, in the context of limited resources, with the exception of certain examples of innovative practice, such as programmes in Kenya [16], Bangladesh [17], Lesotho [18] and Brazil [19]. The central tenet of some of these programmes is a “training-the-trainers” approach; equipping and empowering families with the knowledge and skills to deliver care, while in other cases they relate more to improving family stress and coping through general parent support [20]. Literature from other LAMI settings exploring the provision of services for childhood disability share common themes of meaningful involvement of users and an ethos of family and community-orientated programmes [21–23]. Though research in childhood disability in resource-limited settings, particularly in the area of service provision, is relatively lacking, it is recognised that different ways of working may be required and needs assessment exercises can help establish priorities [24]. Involving both user and provider perspectives in such processes to inform the development and implementation of programmes is essential if they are to be effective, sustainable, and respondent to local challenges [4].

Understanding the lives of those affected is clearly essential. A number of studies in LAMI countries have explored the lived experience of disability for children and their families, yielding important information on the multitude of challenges faced and the priorities for service delivery [20,25–30]. No research to our knowledge has focussed on the experience of caring for children with neurodisability in Malawi, particularly following a more acute brain infection/injury. This study evolved from the author’s clinical observations that such children were often not getting the support that they required, and were frequently re-admitted to hospital or seen in clinics with secondary complications. The authors’ felt that investing in services for children with neurodisability and training health workers (HW) in this area would be beneficial, however given the limited resources; an exploratory needs assessment exercise was required.

The aim of this study was therefore to explore the perspectives and experiences of parents/carers (PC) and HW caring for children with neurodisability secondary to brain infection or injury in a hospital setting in Malawi. The objectives included exploring the patient journey and existing care practices, defining the needs and priorities of families and staff caring for children with neurodisability, and understanding the challenges in rehabilitative care. The findings will inform priorities for development and evaluation of services for paediatric neurodisability, HW training and areas of future research.

## Methods

### Methodological approach

An exploratory approach was used in this study as it is an under-researched topic area, and a qualitative methodology would allow for the investigation of the experiences, perspectives and understanding of PC and HW caring for children with neurodisability [31,32]. Semi-structured in-depth interviews (IDI) were chosen to explore individual perspectives, beliefs and experiences [33], and focus-group discussions (FGDs) were used to generate data on shared experiences and understanding, and attitudes and behaviours sparked through interaction [34]. Validity of the study was increased through the use of triangulation and respondent validation [35].

### Study setting and sample

This study was conducted in the Paediatrics department of the Queen Elizabeth Central Hospital (QECH), Blantyre, Malawi, between June and August 2012. QECH is an urban tertiary referral centre and one of only two hospitals in Malawi with the expertise and capacity to treat children with brain injury and severe neurological problems. Local stakeholders including disabled people organisations (DPO) and paediatrics staff were consulted prior to launching the study, and their involvement informed the study design and materials.

A purposive strategy was used to sample caregivers of children with neurodisability following brain infection/injury, with the aim of including a range of different cadres of HW, and both male and female PCs. Six PCs were recruited from inpatient settings and eight from outpatients. Most children had suffered the episode of brain infection/injury in the previous 3 months (range from 2 weeks to 2½ years).

Fourteen IDIs were completed with PCs, and 10 IDIs and four FGDs completed with HWs (Table 1). Generally, we aimed to use “natural groups” for FGDs (e.g. palliative care team, nurses, allied HW involved in rehabilitation), while also paying attention to potential power disparities and cultural norms. One FGD was made up of nurses who had previously attended a pilot afternoon training session for nursing staff on paediatric neurodisability, allowing the specific opportunity for evaluation of this training.

**Table 1.** Participant demographics.

Participant number	Sex of parent	Age of child	Child’s health condition
Parent/carer in-depth interviews			
PCIIDI1	F	3 years 2 months	Gross motor impairment, abnormal movements, speech, visual and hearing impairment following TB meningitis, multiple strokes, and secondary hydrocephalus
PCIDI2	F	2 years 6 months	Diplegia and gross motor impairment from TB meningitis
PCIDI3	F	9 years	Hemiplegia following traumatic brain injury
PCIDI4	M	6 years	Hemiplegia and ataxia following a stroke
PCIDI5	F	5 years	Gross motor impairment, generalised spasticity, incontinence and speech impairment following traumatic brain injury
PCIDI6	F	8 months	Global developmental delay post febrile illness in neonatal period
PCIDI7	F	4 years 4 months	Hemiplegia, speech and visual impairment, and epilepsy following probable cerebral malaria
PCIDI8	F	8 months	Hemiplegia and focal seizures following TB meningitis
PCIDI9	F	3 years 2 months	Gross motor, speech impairment and epilepsy following cerebral malaria
PCIDI10	F	1 year 6 months	Global developmental delay following cerebral malaria
PCIDI11	F	1 year 1 months	Hemiplegia following cerebral malaria
PCIDI12	F	5 years	Gross motor impairment, speech and swallowing difficulties following TB meningitis and multiple strokes
PCIDI13	F	7 years	Hemiplegia, speech impairment, cognitive impairment, swallowing difficulties following coma (underlying cause unknown)
PCIDI14	M	11 years 7 months	Hemiplegia, facial nerve palsy, swallowing problems, and speech impairment following cryptococcal meningitis
Participant number	Sex of professional	Profession	
Professional/health worker in-depth interviews			
HWIDI1	F	Nursing Matron	
HWIDI2	M	Clinical Officer	
HWIDI3	F	Clinical Officer	
HWIDI4	F	Rehabilitation Technician	
HWIDI5	M	Junior doctor in Paediatrics	
HWIDI6	M	Speech & Language Therapy technician	
HWIDI7	F	Ward Nurse	
HWIDI8	F	Ward Nurse	
HWIDI9	M	Paediatric Registrar	
HWIDI10	F	Occupational Therapist	
Focus group number	Number in group	Professions of those in group	
Professional/health worker focus-group discussions			
HWFGD1	17	Nurses from paediatric malnutrition ward, medical bay, special care ward, malaria ward, outpatients and A + E	
HWFGD2	4	Paediatric palliative care team: nurses, clinical officer	
HWFGD3	7	Nurses who had attended neurodisability training session from malaria ward, medical bay, orthopaedic ward, special care ward	
HWFGD4	5	Other professionals: Rehabilitation technicians, Playladies, Hydrocephalus specialist nurse	

### Data collection

Study materials (IDI and FGD guides, information sheets, consent forms) were developed collaboratively by the study team in Malawi, informed by themes from existing literature, and discussions with DPOs and service providers. IDIs and FGDs were undertaken by a male Malawian social scientist (CM-C), and were conducted in Chichewa with PCs and in English with HWs. Observations and notes were recorded daily in a research journal by A. P. Each IDI and FGD was discussed by the research team afterwards and transcripts reviewed during the data collection period. Saturation was reached when the research team felt that data collection was no longer shedding any new light on the topic under investigation.

### Data analysis

IDIs and FGDs were audio-recorded, transcribed and translated. Transcripts were checked for quality, and data analysed using NVIVO software. Thematic analysis was conducted using an inductive approach where codes and themes were sought in the data. A list of codes was created which was then continually reviewed and restructured during the coding process to reflect emerging ideas [36]. Analysis included comparing findings across participants, grouping codes and linking themes in new ways. The main themes generated in the analysis were presented at QECH in an interim research dissemination. This allowed opportunity for respondent validation and new perspectives on the data to be included, particularly the need to use the data to explore practical solutions to the challenges faced using an assets-based approach.

### Ethical considerations

The study was approved by the Research Ethics Committees of the Malawi College of Medicine and the University of Liverpool (Ethics numbers P.04/12/1204 and RETH000535), and institutional approval was gained from QECH Paediatrics Department. Informed consent was gained from all study participants.

## Results

Our analysis generated six main themes. In all cases, the perspectives of PCs and HWs are integrated.

1. **Perspectives on neurodisability**

### Prevalence and aetiology

HWs discussed the prevalence and significant workload of paediatric neurodisability.

Because, in the past, as I was explaining, I think we would see very very little problems like neuro deficits. But now, I think medical ward is full of patients. HWIDI5

HWs mentioned many causes of neurodisability and felt the disabling potential particularly of malaria and meningitis was underestimated. Understanding about causes among PCs varied and was influenced by religious or cultural belief, health literacy, HWs and community members. Even when aware of the diagnosis, PCs were sometimes unsure how a condition such as meningitis could lead to functional problems like difficulties with walking.

Some of them think that the child has been bewitched maybe, that’s why he has developed that……. They can’t think of the malaria HWFGD3

My worries were in the sense that, since this one started all this from the village alright …. Therefore I saw that maybe there was some magic that had really come in indeed. PCIDI10

### What the future holds

HWs often described the prognosis for children with neurodisability as poor and therefore counsel parents that there was no hope for improvement.

We tell the carer, the mother should have love, because this child, he is going to remain like this… Because although he is growing, but still, there is no change… he will be like this. HWIDI 7

Despite reporting a belief in healing, many PCs were keen for information on what they could expect in the future, and their questions revealed concern and uncertainty, e.g. “Will he walk again? Will he go to school?” HWs recognised that hopes and expectations of PCs were often high, particularly that the child would be “healed” with no residual neurological impairment, and suggested such concerns should be addressed through better communication.

…make them understand as to why the large referral hospital like this one has managed to discharge the patient in that condition. Because to them the patient is not well enough, so their expectation is for them to continue staying in the hospital… HWFGD2

PCs suggested that prayers, exercises, equipment to help with stretching the child’s limbs and medication might help their child’s prognosis, but some felt that “nothing can be done”. A duality between religious belief and acceptance of a medical model of management was sometimes relayed.

Parent/Carer:Prayers of course they could also help.

Interviewer:Mmm.

Parent/Carer:But still these things go together, prayers and the hospital. PCIDI2

The help that I feel is needed is that: if only the drug could happen to be found…. that this child should be given and begin walking PCIDI2

PCs sometimes viewed medical interventions (e.g. a drug) as offering a potential for cure, a view many HWs wanted to guard against, by recommending that any intervention (e.g. dispensing medication) be accompanied by information to prevent unrealistic expectations.

1. **Problems faced by children & their families**

### Main difficulties

The most common problems for CWD from the HW perspective were feeding and swallowing difficulties, motor impairments and immobility, speech, visual, and hearing impairments. PCs focussed on physical disabilities, feeding problems and incontinence (Table 2). HWs and PCs noted that less visible cognitive, behavioural or speech impairments only become evident later.

I think the carer [HW] would have to assess properly, like for the immediate, those that you can see, the complications that you can see. Let’s say, if it is hearing loss, then it is easy to assess here. If it is visual impairment, you can assess here. But the others that you know later, like the behaviour thing, so the time they are… getting out of hospital, you do not know that this child will have a behaviour problem. HW IDI2

**Table 2:** .Problems faced by children with neurodisability.

Problems	Illustrative quotes
Impaired function	
• Motor difficulties/immobility • Feeding/swallowing difficulties • Continence • Visual/Hearing impairment • Speech/language impairment • Behavioural problems • Cognitive impairment	“We had a certain child when she had a disability she was 5 or 6 and when she became 8 years she was becoming… fat so even for the mother to carry her from the mat to a chair it is difficult and I think she was alone … nobody was assisting her. So it was difficult for her to even carry the child from the mat to the chair… She was just doing everything on the mother” HWFGD1 “Because some even find that, they are even maybe wetting the bed, or, defecating on the bed, or, they can’t say ‘I want to go to the toilet’” HWIDI 08 “…these patients with defects like eyesight, hearing, they are just dumped in the community and their school is affected” HW FGD2 “Even when I am looking into his eyes… I sometimes doubt that he is able to see” PCIDI 01 “Although he cannot speak, but I am still able to talk to him. So on my own am able to know that when he is moving the eyes or the head, shows that we are understanding each other” PCIDI01 “…you find that most of the neurological sequelae that we get is kids who come with behaviour changes… sometimes like they become hyperactive, sometimes disorientated….” HW IDI03 “Of which she needs much help on, the one I happen to feel is really a problem…… It is that one of having a head that doesn’t function properly” PC IDI10
Secondary problems	
• Aspiration • Malnutrition • Pain/distress • Pressure sores • Contractures • Poor seizure control	“Choking may result leading to several complications, pneumonia among them, not to mention about death*…* “HWFGD4 “…a number of them they might come back with malnutrition… because of feeding, so skills on food preparation and how to feed the child, what to feed… is very important because many of them will come with malnutrition several times“HWFGD2 “What we do is that we give analgesics, but we would like to know why they are crying? Because they do not stop, they can cry 24 hrs.” HW FGD1 “That’s what is making him at times you could see that he is crying uncontrollably and also becoming troublesome…” PCIDI 01 “my experience shows that these disabled children it’s better to nurse them on a mattress at home because they don’t get bed sores, most of the times, but in addition… in the villages they put them on a mat which is hard so they develop bed sores easily” HWFGD1 “They have no power, or when she wants to crawl then these limbs remain behind and she bends the arm like this…” PCIDI 11 “…drugs to prevent the seizures. Because if they don’t do that as well the seizures will continue, and they will still be coming to the hospital each and every time.” HWIDI 09
Activity/participation	
• Dependence/lack of independence • Unable to attend school • Lack of social integration • Unable to work/contribute to family	“The difference was in a way that: since she was not able to sit down, she was not able to do anything, she was always on the back…… Eeh, therefore this really used to be hard for me, it was really a problem” PC IDI 10 “It’s a very big blow because it’s like they are having a child, who is like, maybe could be ten years, but to them it’s like a one day old…. he needs to be fed, he needs to be bathed, he needs to change nappies now and then, you know it’s a very big blow.” HW IDI05 “Since it looks like his head is not functioning well…. Sometimes he gets lost on the way. Therefore with his inability to speak…. even his hands have not reached a point of maybe taking a pen and write….” PCIDI 13 “…since the child is immobile, is not walking, is not talking, and this one is an older child because is 6 years, maybe before she or he got sick was able to play with other friends and could do everything on its own, the child, so we advise the parents or the guardian never to isolate the child…” HWFGD 3 “the problem that I see as being big is that of coming back to normal like in the past so that she should start doing all the work she used to do” PCIDI 14
Burden of care	
• Gender issues • Family separation/ marital issues • Physical burden • Opportunity costs • Financial issues • Care of siblings • Maternal stress/ depression	“Yes, therefore I as a parent, his mother, I have accepted to help my child in everything” PCIDI03 “The fathers are not really keen to help the mothers in caring these children with disabilities…. Yeah, and you find that most, most fathers, when they have seen that now the child has become disabled, then mmm, they are not happy with that” HWIDI 10 “Some people they get divorced because the mother is too busy taking care of the …. depending on severity of the disability and how long it has stayed, the mother is taking too much time taking care of this child and is maybe ignoring the husband” HWFGD3 “The other area which I see to be a problem for the mothers is, if this child has been walking, doing all sorts of activities, and now here they are, grounded. And if that child is growing, it will be a burden to the mother, to the guardian. “HW IDI 1 “So it becomes difficult for me to do other chores, for example I have not yet started cultivating the fields….” PCIDI11 “I think financially that’s where it comes in, because, money is everything I should say, because you need to give them the right food, you need to come with them to the hospital when they get unwell…. yeah, they need to take them to school.” HW IDI 3 “…if they have other children at home they are thinking ‘Oh! If this time if I go again I will also stay longer again, what will happen to the other kids?” HWFGD3 “when they are small babies or toddlers, they don’t seem to be very much depressed, but when the child is growing up… now the care has to increase. And even when you are growing up, you still, the child needs to feed more, so the time for them to spend feeding these children, they get tired, each and every time.” HWIDI09
Neglect & abuse	
• Left alone/locked up • Not cared for • Not fed • Not taken to health services when unwell	“….they are just being kept in the home… They are locked there and people go out to work. They don’t care for them.” HWFGD2 “You know they need love… because there are some, I was doing some follow-ups, some time ago, and I find the child who they are dumping in the house, doing their business or doing other things, staying the whole day without eating…. so I think they need love.” HW FGD1 “…there are some who just dump them in the house, without feeding them, without caring them…” HWFGD1 “Most of the patients when they are discharged with such complications, they just close them in the house, even if they fall sick they don’t even bother to take them to the hospital.” HWFGD2 “….sometimes because of the stigma in the society they would just leave it like that, after all he is already sick so…” HW FGD3

### Burden of care, opportunity costs and gender roles

Mothers are seen as primary caregivers but some report assistance from husbands, family members, relatives and church groups. Both fathers interviewed played active roles in caring, though this appeared out of the ordinary. One HW discussed difficulties in engaging fathers in caring for CWD, and that “good fathers” in this situation is rare (*One! In a thousand! - HWIDI10*).

Although mothers normally shoulder the burden, HWs and PCs acknowledged the impact on the whole family, particularly children with physical disabilities due to the physical burden of carrying a growing child and the limitation on caregiver’s activities. This notion was described as being “grounded”.

It’s just so difficult, let’s say, myself, I was moving freely at home… and then, the child was maybe independent, or else they were able to leave that child, because it was able to walk or able to do much of the things by himself or herself…… and then develops that condition, it means you are grounded. HWIDI8

PCs reported that caring for their child left little opportunity for household chores, social activities, income-generating schemes, and caring for other children. Financial costs of caring for a CWD, particularly the costs of transport were also raised. HWs suggested that the burden of care can lead to depression and burnout in the mother, acting as a potential risk factor for abuse and neglect.

Because sometimes they, they can give up, and you find the child is not just, getting any better, because they have that expectation that the child will get better. But the child is not getting better, and then they can, they may stop giving the support. HWIDI02

### Neglect and abuse

The risk of neglect and abuse of affected children was an issue strongly raised by HWs. Risk of neglect was not substantiated, though not explicitly explored in PC interviews. Mothers were tender in their care but occasionally shared concerns that neglect could be a possibility.

All I can do is just accepting that’s all, once I happen to get home, I will take care of him, just as a small child, just the way I used to take care of him when he was a baby…… PCIDI1

I am actually giving him more love than before so that he should not be worried that I have abandoned him because he is now lame. PCIDI1

1. **Barriers to care**

### Long-term care not a priority

Multiple barriers impeding the longer term care of children with neurodisability (Table 3) included health system, service provider and wider context factors, with barriers existing at every level. The strong prioritisation of acute illness was particularly evident with shortages of resources (space, equipment, medicines, HW) and few trained rehabilitation staff. Many HWs had more knowledge and skills than they realised but lacked confidence. Missed opportunities were evident and many reported that the best use of resources is not often achieved. Factors playing a role included HWs lack of awareness of services, poor communication, lack of standardised assessment procedures, unclear referral pathways, and poor availability of community services.

**Table 3.** Barriers to rehabilitative care and illustrative quotes.

Barriers	Illustrative quotes
Acute illness is the priority	“Mostly in our ward we only give more time to, like the patients that are very sick because of the shortage of staff.” HWFGD3
	“Because here they come because of their acute problems that they have at that particular time…. so if it’s malaria, then it’s just malaria you treat….” HWIDI02
Loss of interest/ hopelessness	“….we just focus on the curative measures but whenever this child has been disabled nobody is interested what is there afterwards” HWFG2
	“…once you have controlled the distressing symptoms, still you feel that this patient, let us see, let us just discharge them…” HWIDI01
Lack of time, huge workloads & staff shortages on the wards	“…the advice is not all that up to the standards, because our department is so busy, and maybe they don’t have enough time to sit down with the guardians…” HWIDI01
	“Mostly, I think because the nurses are so overwhelmed with putting up drips, giving out medication, whatsoever….” HWIDI10
	“At times, you want to do something to the mother or to speak to the mother, or do something to the child but you can’t do it because of shortage of staff…” HWIDI08
	“…do nurses have time to listen? Because yes they would love to talk about all this and do this and do that, but have they got time to listen?” HWFGD3
Lack of rehabilitation professionals	“…we are just a few of us working and there are a lot of children who need us… who needs our services, yeah that is the main challenge that I see.” HWIDI04
	“because of lack of the trained personnel in other disciplines, like the occupational therapist, physiotherapist, or the social workers, you will find that mainly like in our country we opt for multi-tasking, where a single discipline is given all those tasks to perform and eventually this particular discipline becomes overwhelmed.” HWFGD2
Lack of space/equipment	“one challenge is also equipments, yeah, those patients they need like standing frames, corner seats, and other equipment that may help, even wheelchairs.” HWIDI04
	“…we don’t have, we can’t give these kids in our hospital, we don’t have no place where we can keep them for a long time…. like how we do in the malnutrition” HWIDI07
Lack knowledge/ training	“Yeah, I think, the nurses themselves we don’t have knowledge about it. We just dish medication then we go…” HWIDI08
	“I feel like there is lack of information to the nurses to understand…what actual type of kids are left in there.That’s why I said I find a lot of kids being fed lying in bed” HWIDI06
Low HW confidence & ownership	“…I don’t know whether it be fear or how they can approach, they don’t have enough knowledge how they can handle such kind of a situation.” HWIDI01
	“….you can’t, you don’t know what to say to the, to the mother or the father or the relatives of the children” HWIDI08
	“…they [Umodzi team] come and do, they do proper counselling. We also do counselling, but I think they do it in a better way…” HWIDI03
Stigmatising attitudes of HWs/guardians	“this cerebral malaria, had you came earlier, we would have helped you. But the problem is you came very late, and now look now the child, brain is damaged.” HWIDI05
	“…because once that child is disabled, illiterate people, they will not even think about the future of that child…. yeah, it ends there” HWIDI03
	“….they won’t like other guardians or parents to be looking at them, like some kind of stigma”. HWFGD3
Focus on physical disability	“…it‘s more physical, physical, physical…… they miss a lot on the learning part, the mind part.” HWIDI10
	“We don’t refer them anywhere…. Usually we just take care of the acute symptoms they come with but the behavioural thing we can’t do anything about.” HWIDI02
Failure to use available services	“[they] are being discharged direct from the ward to home, while there is a department here. But that could be because somebody may not have the information” HWFGD4
	“Because you know, here we complain about human resource until God knows, but we can use those playladies and just tell them what they can do with the child” HWIDI10
Poor awareness of services	“…there is also another challenge that programmes do not talk to each other” HWFGD2
	“Maybe there are organizations which are looking at issues of disabilities but at a community level itself I don’t know. Maybe they are there but I am not aware” HWIDI01
Poor access to services	“… worst still, according to our locations of the health facilities within our communities, the radius of health facilities are just too far apart where there are no services of rehabilitation when they have been discharged. As a result they are just left out in the village, isolated, nobody is there to help” HWFGD2
	“They are coming from poor families so sometimes means they complain it is far from the hospital, they are not able to go to the centres, to come to Queens…” HWIDI09
Poor recognition of the issue	“it is just unfortunate that quite a lot of resources are poured towards managing malaria as here is the number one killer disease, but nothing was thought of what maybe….….happens after those people have survived the condition” HWFGD2

### Attitudes and stigma

A paradox emerged with the frequent use of the phrase “there’s nothing we can do”, then often within the same interview suggestions of simple ideas to help.

…because of the disability which is there, as a society, we lose, maybe I don’t know whether I can say, interest to the patient… because we don’t know what really can we do… HWIDI1

It’s just simple things that you think they will not make a difference, but they can. HWIDI8

The PCs and their children faced stigma from HWs as well as from other families in the hospital and community members. Certain HW’s suggested that some parents did not accept the responsibility of caring for their child.

That’s why we see some parents when they have been discharged, they go home and they like to lock their children in a bedroom and they go away, just because the child is not doing anything, so they think that when they attend the child, they can’t work anymore; hmmm! They are failing to work, to go to the garden whatsoever, so instead they lock them in the house, so that’s why I suggest they need to be told thoroughly well, the responsibility of their children. HW FGD4

1. **The importance of communication**

The vital role of communication and counselling of families was the strongest recurring theme throughout the data.

…. really it’s the acceptance part that has got to be worked on, to say make the mother understand and then maybe if she understands the problem will be able to accept…… HW FGD4

There was a compelling sense from HWs that little could be achieved until parents understand and accept their child’s condition, with well-informed families better able to care for, and less likely to neglect or abuse their child.

I have seen some kids that have gone to their villages and they develop complications because there was nobody managing them or the parents were not trained a little bit, they were not told what to expect HWIDI6

HWs described that better communication would ensure PCs were empowered, competent and confident to care for their child at home, and able to challenge misconceptions and destructive advice from the community.

If parents they had such information, I believe they would be able to be proud mothers, to help their kids… HWFGD4

Health-Worker: The community may give them information maybe to seek help from other source, maybe from African Healers or maybe using other remedies that may not bring actual help to the patient. So if you give them the right information in the hospital, when they go out there they examine every other information that they receive from their friends…

Interviewer: They are equipped?

Health-Worker: yeah! And they make a very good decision. HWFGD2

### Challenges to communication with families

Adequate time for counselling, a lack of HW knowledge and confidence, and the ability of PCs to understand information about their child were seen as the main barriers preventing good communication with families.

…most parents, most of the guardians we have in the hospital, their levels of understanding usually are not the levels we could want them. And it might as well be on our part we might not have all the time or even the expertise to explain… HWFGD4

The fine balance between managing expectations and maintaining hope was considered a communication challenge by many.

The other thing is uuum, managing the expectations of the parents, because some parents will show up with great expectations, because I think they have been told this place is a centre of excellence, and they have too much expectations… maybe the child will walk, speak, whatsoever, and then after you do an assessment you have to counsel them. Sometimes you have to manage their expectations, because when you don’t manage it well, you either give them false hope or you either lose them, you see. HWIDI6

### Right to information

HWs perceived that PCs saw acquiring information about their child’s condition as a privilege rather than a right; hence, it was unusual to get requests for clarification.

Others it is just lack of acceptance. Others when you explain to them they seem to understand, but if someone comes and ask them what’s wrong with your child, they say ‘I don’t know’. HWFGD3

On the other hand, almost unanimously, PCs felt they had not understood or been adequately informed about investigations, diagnosis or management of their child’s condition. Although some PCs recalled being told about various aspects of diagnosis, prognosis and caring for the child at home, many were left with unanswered questions. HWs postulated that poor understanding contributed to PCs unrealistic expectations, and consequently a lack of continued rehabilitation in the community.

[Sighs] they did not tell me anything, I was just receiving treatment…… Eeh, but they did not tell me anything. PCIDI10

HWs reported that they normally provided information on diagnosis, management, investigations, prognosis, and how to care for the child at home but this needed tailoring to the educational level of the carer. Explanations were sometimes considered too “biological” or too basic for carers.

This was when they came and said: it is the brain……… The brain has gone out of order and is worn out, I don’t know whether they were saying: it is worn out, I did not understand that either. PCIDI12

….Not just to be told that the brain is damaged, because to me if someone tells me ‘your brain is damaged’ there is no hope in that. The other parents who feel the whole brain is damaged, they come to us and say ‘I was told the whole brain is damaged’, I say ‘c’ mon your child is still alive! The whole brain is gone?!’…. So the information we give at times confuses these parents and they say ‘why should I bother?’ HWIDI6

Caregivers also had a multitude of ideas about how communication in this area could be improved (see Theme 5).

1. **Ideas for improvement**

### Standards of assessment, care and follow-up

“Expert” HWs (rehabilitation and palliative care staff) highlighted the need for every child admitted with significant brain injury/infection to have a thorough assessment for sequelae, referral to appropriate services before discharge home, and at least one follow-up appointment.

So what we need to do is to make sure that at least that journey is followed in the first place. Because there is that evidence that every patient who suffers from complicated malaria, it cannot leave him or her without the sequelae. So the emphasis on that is to be on the lookout for such symptoms.… HWFGD2

It was suggested that having a standard of care for all children with neurodisability, with standardised assessment tools and protocols, could help HWs approach the management of these children systematically. HWs emphasised that tools must be clear and simple and must help the HW to decide on actions to take, rather than tokenistic paperwork that adds workload. HWs proposed that parents should also be taught to monitor for potential complications that may not be obvious in their child at time of discharge, and be informed how and where to seek help.

### Dedicated leadership, team, space and equipment

HWs highlighted that human and material resources were often a limiting factor, so achieving improvement in rehabilitation services required leadership, a suitable team, designated space and appropriate equipment. HWs raised a specific need for social workers to be involved in the care of children with neurodisability, particularly to address concerns about child abuse and neglect.

### Effective use of existing services and resources

One HW highlighted strongly that in a resource-limited setting, with funding for sufficient HWs and equipment unlikely, it is first important to ensure that existing services are utilised. It was suggested there is a need to raise awareness and ensure HWs have an appreciation of the existence and roles of services available. HWs also emphasised the timely use of rehabilitation services; with intervention and intense input in the early phase seen as essential.

…I’ve always emphasized on early identification and early management. Here at Queens, those kids I have seen maybe 3 or 4 times during the acute stage, I have seen them walking, I have seen them talking again. Why? Because we intervened before the brain starting to learn the bad movements HWIDI6

### Counselling, communication and parent training

What HWs felt that PCs wanted to know about their child’s condition and what PCs actually wanted was often disparate (Table 4). HWs saw good communication as needing a sense of empathy and love, and the time for “sitting down” with the parent/carer. They also highlighted that good communication needed to be extended towards the wider family and community, and should address issues of stigma and the support needed when children go home.

I think listen to the mother, whatever she is telling you, have some time to listen to her, that will ease her HWFGD3

Encouraging them, that though the child has developed that condition, that doesn’t mean it’s the end. HWIDI8

**Table 4.** Caregiver’s ideas about improving communication with families of children with neurodisability.

Content
What parents/carers want to know
• Diagnosis – explanation of what exactly is wrong with their child
• Why a problem in the brain affects certain functions e.g. walking
• Investigation results
• Treatments
• What will happen in the future in terms of recovery of specific functions (e.g. walking) and participation (e.g. attending school/work)
• Whether it will it happen again (e.g. with future malarial episodes)
• How to help their child’s recovery and function
What health-workers think parents/carers need to know
• Diagnosis
• Prognosis
• Management plan
• How to ‘handle’ the child including correct positioning and turning the child in bed
• Basic nursing activities
• Advice on feeding and nutrition
• Physiotherapy exercises
• How to use the ‘CP’ (Cerebral Palsy) chair correctly
• Purpose and administration of medication
• How to help the child sit and walk
• How to stimulate speech and hearing by talking to the child
• Importance of social integration; allow the child to be with other children
• How to monitor for potential sequelae of brain injury that may not be evident at time of discharge e.g. delayed attainment of developmental milestones
• Follow-up arrangements and available services to support rehabilitation
• General health promotion; seek help early for illness in their child, use preventative measures e.g. mosquito nets
Format
• Start from the day of admission, don’t leave it until the day of discharge home
• Allow sufficient time and privacy
• First build good rapport
• LISTEN to the Parent/Carer’s concerns
• Carry out with a sense of empathy and love
• Encourage and motivate Parent/Carers
• Understand the subject you are talking about
• Explain reasons behind recommendations e.g. feed the child sitting upright to reduce risk of choking or aspiration
• Repeat and reinforce key messages
• Make the sessions interactive, practical and fun
• Adapt for the Parent/Carer’s level of education and literacy
• Use materials e.g. pictures/leaflets to support not replace counselling
• Involve fathers, families and communities
• Make sessions regular/ongoing, not one-off
• Consider group counselling sessions and peer support methods

Overall, caregiver’s suggestions for improving communication practices (Table 4) focussed mainly on attitudes and formats for counselling. HWs reported a strong desire for training in counselling skills.

In terms of how best to deliver information to PCs, HWs discussed the limitations and benefits of leaflets in this setting. Some HWs felt that such materials could be helpful for those PCs who were literate, but less so for the many PCs unable to read, unless purely pictorial.

Most of our, mothers who are in dire need of this information, they are illiterate. Because even I find it, even with the few learnt people, you know the reading culture is not there. So most of the times, people have found it hard for us in this part of the world to use posters like this and things like that. HWFGD2

It was felt they could be used as a reminder for what had been discussed while in hospital, and may also help to disseminate information to the wider family and community, e.g. siblings who may have higher literacy levels. HWs emphasised that such materials must be used in addition to, not instead of counselling, and should be bright, colourful, clear, not too wordy, and with culturally appropriate pictures. Creating generic written/pictorial materials was felt to be challenging, given that each child is unique with different impairments and functional limitations. A number of suggestions were made including the main conditions causing neurodisability, types of disability, available services, nutrition and feeding, play and stimulation, handling, positioning and social integration.

PCs feelings about leaflets were equivocal. Many reported they would be comfortable with a verbal explanation, whilst others felt additional materials could be useful.

They can just speak, and I can understand. PCIDI10

The pictures could be needed [laughing]…… I should be looking at the pictures then you can be explaining to me.’ PCIDI11

### Peer and group support

Many HWs suggested that informing and empowering PCs would be best achieved through peer support groups and group counselling, perhaps aligned to a follow-up clinic or outreach service.

Let them come one day, to discuss their fears, and they can also come up with ideas on how they can take care of their children. HWIDI10

Such groups were seen to provide the psychosocial support that HWs recognised is important in helping PCs cope. HWs felt that groups may also be a way of raising awareness and sharing information if family members were invited.

…maybe to have, what I can say, whether a clinic whereby maybe these mothers can be meeting and be supporting each other, they can share ideas. And also I’m looking at issues of maybe, during that time, it can also be like, a relief for them. HWIDI1

1. **Training for all comes first**

### Barriers to training

Most HWs reported the key barriers to training in paediatric neurodisability were funding and opportunity. Other barriers included the range of information to be covered and differing training needs and background levels of skill and experience among different cadres of HW. Some HWs felt that lack of prioritization, motivation and leadership prevented training in this area.

I think I wouldn’t say it’s money, no, it is not funding. Like to be trained in basic things here at the hospital? I think it’s the motivation, or someone to take it up, I don’t know, there is no-one to take it up, to say this is my project I want to do it. Because to me, organizing people to meet in a hall, maybe you just need a small amount of money for their lunch sometimes or a snack, they get the training, that’s it. It doesn’t need too much funding but maybe there hasn’t been nobody to initiate it, you see. HWIDI6

### Ideal format and structure of training

HWs felt that the potential range and volume of content means that training requires sufficient time dedicated to it. HWs were keen on the inclusion of practical skills (e.g. assessing swallowing) and seeing real cases through bedside teaching. HWs generally felt that it needed to be spread over a number of sessions, and followed-up by regular meetings providing the opportunity to consolidate learning, discuss and reflect on cases and work through real problems.

## Discussion

This study explores the experience and perspectives of those caring for children with neurodisability in a hospital setting in Malawi. The WHO World Report on Disability recommended that increased research is required on the type and quality of rehabilitation services available, as well as unmet needs and barriers faced, incorporating user and health care provider views [4]. This research has worked towards this goal and has provided rich data to enable us to consider important aspects for developing services and providing training for HW in this area.

In our study, we have demonstrated how caregivers can generate a multitude of useful ideas, and envisage that listening and incorporating such views will make interventions less likely to fail at the first hurdle. Ideas generated by those who work on the ground or who require such services are likely to be inherently more responsive to the needs and challenges of the local context; indeed, it is noteworthy that many of the suggestions are already aligned to the level of resource available.

### The experience and impact of caring

This study demonstrates the high burden which some families’ experience in caring for CWD. Similar themes around indignity, exclusion, pain, household poverty and hunger are reported in studies in Malawi [25] and Kenya [26]. Collective evidence suggests that the human experience of caring for a child with disability has some fundamental similarities across settings. The challenges may include wide impacts on the family, on carer’s health and well-being, financial and opportunity costs, stigma and discrimination, and services that are inaccessible or insufficient to meet needs [37,38]. It is likely that many of these challenges transcend national, cultural and economic boundaries. Children with neurodisability in low-income settings have the same rights and needs, which must not be forgotten when resources are scarce.

However, specific contextual issues do exist and need to be taken into account, such as the intertwined nature of disability and poverty, where, in settings like Malawi, the consequences may be more extreme. The impact of caring for a child with neurodisability on household productivity was clear in our study; not just the effect of the primary caregiver being “grounded” and unable to work, but also around potential lost future productivity of that child. In families with many members to support and limited resources, it may be that the child with neurodisability is the last mouth to feed. The risks of malnutrition and neglect of children with disability partly reflect the setting of significant poverty. This highlights the need for services to be developed alongside programmes of poverty reduction and integrated into health services, particularly those for malnutrition [39,40].

### Strength and resilience

Despite the innumerable difficulties in caring for CWD in this setting, there is strength and resilience both from PC and HW. Caregivers do not experience their roles as totally negative, and recognise that even “simple things can make a difference”. It is suggested that these positive perceptions play an important role in coping with the child, the disability, and the difficulties associated with it; and may also help the family function as a whole [41]. Other mechanisms, such as support and belief systems may also be employed to deal with the challenges faced [29]. Interventions which support, empower and strengthen the capacity and resilience of mothers are seen as essential for the well-being of CWD [30]. This corresponds with the priority given in our study findings to improve communication and support of caregivers.

### Communication with families

A focus on improving communication between HW and families does not have high costs attached to it; yet enabling it to be done well requires time, training and processes of evaluation. An intervention would aim to help parents and families understand their child’s condition, and empower them with appropriate skills and knowledge for caregiving activities at home. Other studies have raised concerns about how literacy skills can affect carer’s in fully understanding health information. The emphasis for health care providers is therefore to communicate in ways that are congruent with mothers’ knowledge, abilities, and style and pace of learning, to enable confidence, skills, and realistic expectations in parents [28]. Our data highlight the particular difficulties in managing expectations and information needs of carers for their child with a disability.

The data illustrate that many parent/carers employed aspects of a personalistic explanatory model when discussing their child’s condition, with a belief that all misfortune, disease included, is explained in the same way; with illness, religion, and magic inseparable [42]. A duality or conflict with medical rhetoric was raised and there was concern about how this may impact upon engagement in ongoing care and support of CWD. The dichotomy itself may reflect Western-based theoretical orientations, therefore exploring beyond such a dichotomy is important if we are to avoid perpetuating a parent-deficit perspective [43]. However, the role of HW in offering a biomedical explanation of the child’s condition could potentially reduce the sense of shame and stigma felt by families, and feed into a disability rights perspective.

### Wider context and implications for service development

This study reinforces the need to consider the wider context when working with families of CWD, and the importance of aligning with the principles of the International Classification of Functioning, Disability and Health [44], and the UN Convention of Rights for Persons with Disabilities [45]. Programmes of rehabilitative care and support should be designed in keeping with such frameworks, using a bio-psychosocial approach that considers the care-giving environment as well as the caregiver’s needs. Overall, there needs to be a shift to a family and community-centred focus; short-term programmes that focus solely on the child, or provision of adaptive equipment, or one single aspect of disability may not fully take this into account.

Improving human resources and training were key priorities identified by this study. Training personnel in low-income settings can be complex as the absence of specialist practitioners and inadequacies in health systems is often present. As suggested by caregivers in our study, training non-specialist and community-based workers can help improve access to services [4]. Specific ideas include the creation of simple guides for HW management with accompanying training, building on schemes created elsewhere [16,17,19,46]. A parallel focus on training and empowering parents in simple therapeutic strategies is important [47]. Providing accurate information about causes and prevention of impairments, the realities of a cure, and support for those carers is also essential [48]. Exploring the potential of peer support and group-based interventions was an important suggestion from caregivers, and one that has seen success in other areas [49]. Improved communication and understanding of disability issues among family members and community groups may encourage people to provide respite through physical assistance or social and emotional support for carers, help tackle the social isolation associated with having a child with a disability, and encourage positive attitudes towards CWD. Opportunities for carers of CWD to meet could also enable them to share experiences and skills and develop creative ideas for additional local support strategies [50].

### Practical impacts of the project

Our research has now acted as a trigger for change and a starting point for improving training programmes for nursing staff as well as Health Surveillance Assistants in the community. Links have been made with programmes targeting malnutrition in children with disability [39,40] and signposting for families of children with neurodisability involved in follow-up studies of cerebral malaria. An assessment tool and a directory of services have also been created for HW in Blantyre to use when managing children with neurodisability.

### Limitations of the study

The study was conducted at the facility level, therefore only sampled PC who had succeeded in accessing health services for their children. The views and experiences of those who have not reached hospital services may well be different and would require a community-based study. We also had limited involvement of fathers, but the study highlighted that important gender differences may exist in this area.

## Conclusion

A range of family, health service and wider context factors affect the care and support of children with neurodisability following brain infection or injury in Malawi. Caregivers report significant challenges and wide impacts on the family. Perspectives on paediatric neurodisability vary between HW and PC and should be taken into account. Multiple barriers to care exist at all levels, but caregivers had numerous ideas about how challenges should be tackled, with a focus on improving communication a key priority. The impetus must now be on taking take these ideas forward to interventions that improve the care of children with neurodisability in such settings.
